# Food Composition Databases: Considerations about Complex Food Matrices

**DOI:** 10.3390/foods7010002

**Published:** 2018-01-01

**Authors:** Stefania Marconi, Alessandra Durazzo, Emanuela Camilli, Silvia Lisciani, Paolo Gabrielli, Altero Aguzzi, Loretta Gambelli, Massimo Lucarini, Luisa Marletta

**Affiliations:** Consiglio per la Ricerca in Agricoltura e l’Analisi dell’Economia Agraria-Centro di Ricerca CREA-Alimenti e Nutrizione, Via Ardeatina 546, 00178 Rome, Italy; alessandra.durazzo@crea.gov.it (A.D.); emanuela.camilli@crea.gov.it (E.C.); silvia.lisciani@crea.gov.it (S.L.); paolo.gabrielli@crea.gov.it (P.G.) altero.aguzzi@crea.gov.it (A.A.); loretta.gambelli@crea.gov.it (L.G.); massimo.lucarini@crea.gov.it (M.L.) luisa.marletta@crea.gov.it (L.M.)

**Keywords:** Food Composition Databases, composite dishes, analytical data, calculation procedure

## Abstract

Nowadays, many countries have their own national Food Composition Databases, whose continuous updating allows the inclusion of a large number of foods, reflecting the food habits of the population and the growing number of foods on the market in the best way possible. Therefore, particular attention should be directed to the study not only of individual foods or food components but also of the nutritional characteristics of dishes, meals and diets, as they are really consumed. Recently, a reviewed sensitivity in Europe towards the implementation of standardized procedures for generating reliable composition data for composite dishes has been carried out. Although direct chemical analysis is the most accurate method to determine food composition, the nutrient content of complex matrices and composite dishes is often calculated from the nutrient contents of the individual ingredients, considering the different thermal treatments and using some preparation factors. In this context, this paper aims to give an updated picture on Food Composition Databases; in particular, their application regarding complex matrices is examined together with the need to optimize their calculated nutritional values. Results obtained by this calculation should almost always be observed as approximations.

## 1. Food Composition Databases

Food Composition Databases (FCDBs) contain nutrient composition, energy and/or other bioactive components of foods largely consumed by a population [1,2], mainly simple foods and non-cooked processed foods. These databases are used to make a series of interventions, including:

(1) assessing the dietary intake of individuals or groups, carrying out epidemiological studies and clinical research; (2) formulating diets at the individual and/or population level; (3) formulating agri-food policy in relation to public health and educational-information and (4) supporting industrial and handicraft companies for their products’ nutrition labeling [1,3,4,5,6,7,8].

The FCDBs also provide the basis for defining dietary reference values [9] and for formulating Guidelines for healthy food and nutrition i.e., for Italy “Linee Guida per una sana alimentazione italiana” [10].

Nowadays, a lot of countries have their own national Food Composition Databases, whose updating should include a large amount of food, by reflecting the main nationally consumed foods and food habits of the population. The level of certain nutrients in some foods available worldwide varies greatly between countries, because of differing cultivars, soils, climates, agricultural practices, processing procedures and analytical methods, as well as many composite dishes with the same name show different characteristics between countries. This is the main reason why data from other countries cannot be used in all situations and why it is essential to develop a national Food Composition Database.

Currently, the FCDBs include different typologies of foods: simple, processed, traditional or “innovative”, certified and local foods, as well as those originating from other countries that have become of more common use; the availability of data depends mainly on economic resources.

Considering the growing globalization of the food market, national Food Composition Databases are addressing to produce, collect and present data in a standardized format in order to “speak a common language” which allows to compare data from different national Databases and use them interchangeably to collaborate between countries.

This need has been carried out in several projects and led to the development of several co-operations including INFOODS—International Network of Food Data System, COST Action 99, “Project Committee–Food composition data” (CEN/TC 387), European Food Information Resource (EuroFIR) Network of Excellence. The latter projects, at European level, have produced a consensus document on standardized procedures for production and compilation of FCDBs and developed a European Food Data Platform: FoodExplorer^TM^ tool (http://www.eurofir.org/foodexplorer/login1.php) [11]. The EuroFIR activity is currently being supported by the Non-Profit Association EuroFIR AISBL and now it offers a set of tools for the exchange and information of validated and standardized data: Food Explorer, Food Basket, eBasis and PlantLibra Database [11].

## 2. The Importance of Food Composite Dishes and Food Preparations Data in Food Composition Databases

Following the evolution in recent years of nutritional science, the study of the potential beneficial role of diet is one of the most topical and controversial. The health status and in particular the risk of certain diseases has been closely related to the diet which, in addition to the concept of correct and balanced, has also been enriched by functional diet (WHO). Research on interactions between single food components and/or between the various ingredients of a composite dish, has a significant role and amplifies the meaning of the “food synergy” as fundamental unit: a concept for understanding the relationship between nutrition and health [12].

It is emerging that experimental and epidemiological studies should consider the “total diet”, as an important variable in risk assessment and the “total quality” of what is consumed to address towards better choices, more consistent with the specific individuality, with the aim to positively influencing physical, psychological and social well-being [13,14]. Therefore, particular attention should be directed to the study not only of individual foods or food components but also to the nutritional characteristics of all foods in the generally consumed forms of the whole diet.

To date, the need for more nutritional data on cooked foods and composite dishes in FCDBs has emerged, beside a marked gap in their composition information. At the time, there are still too few studies in literature that take into account the complex food formulations (ingredients, preparations, cooking method) on nutritional properties of composite dishes [15,16,17,18,19,20].

Recently, this issue has drawn attention in Italy and has led to a reviewed sensitivity in Europe towards the implementation and standardization of the procedures for producing new and reliable data for composite dishes [17,21].

In this regard, in the last years research strategies in Europe have been addressed towards the study of the nutritional composition of traditional dishes commonly consumed throughout European Countries (EuroFIR project-European Food Information Resource Network of Excellence) [18] in order to know what we eat, in comparison with dietary recommendations and to include their nutritional characteristics in each national FCDB. In this project, efforts in developing procedures for defining and establishing a standardized approach of study (description, consumption, selection, collection, preparation, references, analytical approach and/or calculation) have being carried out [11,19,21,22,23,24].

## 3. Nutritional Composition of Composite Foods: Comparing Analytical and Calculated Data

The increasing consumer’s attention on knowing about what we eat and the mandatory of nutrition labeling by the industry, has increased the need to produce more complete composition data [25,26]. On the other hand, the poor availability of data for specific food formulations, often on the market for a short time, clearly indicates the need to study alternative methods to analytical methods to know their nutritional properties.

Although direct chemical analysis is the most accurate measure of nutrient composition, it is out of reach for high cost, its long and time-consuming analysis and requires also complicated procedures, adequate equipment, specialized personnel. Furthermore, the abundance and the variability of preparations of the same recipe in the Italian diet, such as the continued presence of new food products on the market, increase the difficulty to analyze most of the Italian consumed composite dishes. For this reason, often the characterization of nutrients and the energy value of cooked foods and especially of complex food matrices should be obtained indirectly by specific calculation procedures. In recent years this topic has been very discussed and nutrient values of complex matrices and composite dishes have often been calculated based on the nutrient content of individual ingredients, considering the different preparation and thermal treatments and by using some correction factors [24,25,26,27].

Calculation methods that provide the composition of multi-ingredient foods are different (Infoods method, British method, Yield factor method, Retention factor method, Method used in EPIC-European Prospective Investigation into Cancer and Nutrition, USDA method, etc. [28,29,30,31,32,33,34,35] in major part of cases based on the nutrient contents of the ingredients (values taken from national FCDBs or imputed from other databases), the amount of each single ingredient in the recipe, information on the cooking techniques (time, temperature, utensils, etc.) and the sensitivity of foods and nutrients to thermal treatments.

The various calculation methodologies can be corrected for preparation factors: loss or gain in weight, usually considered as yields and nutrient changes, usually considered as retention factors [21,22,36,37,38,39,40], providing therefore “more or less representative estimates” of the chemical-nutritional characteristics of the recipe under consideration.

Hypothetically, nutrient retention factors should be available for each type of food product and depending on the specific method of cooking [41].

Several studies [36,42,43] have shown that the retention of nutrients, in the same cooking conditions, for some foods is similar. For example, for different types of red meat roasted the same retention factors are applied to different foods but belonging to the same food group, or subgroup, when subjected to similar heat treatments. Similarly, for complex food matrices [41], retention factors are considered similar to those of the main ingredient and are therefore “imputed” to complex dishes.

For this reason, studies aimed at comparing calculated data and data obtained from chemical analyses are needed to optimize and asses the correction factors (retention factors and yield factors).

Nowadays, several research studies are being carried out on the development and validation of the calculation method to know the nutritional contents of complex matrices. The comparison between analytical and calculated data allows to evaluate the quality of calculation procedure; its results can be considered a valid alternative to analysis or only a rough estimate. Calculated values are not suitable to all uses and more attention must be paid to nutrient intake studies, especially for micronutrients, which exhibit greater variability, because they are present in very small content and are more sensitive to preparation and thermic treatments [17,44,45,46].

In addition, reliability of recipe calculations depends on several factors i.e., the presence of data missing, the lack of specific retention factors, the use of national food composition databases [22,47]. The final result should always be carefully evaluated and considered more or less reliable depending on its use. For instance, with the Regulation (EU) No. 1169/2011 [24] for food labeling, the use of calculation procedure to formulate the nutritional labels of processed products, was consented as possible alternative to direct chemical analysis. In fact, the mandatory allows a discrete margin of tolerance of the values.

On the basis of previous actions [37,48], a single procedure was discussed, compared and validated under EuroFIR project (www.eurofir.net). This calculation procedure considers the application of a yield factor at the recipe level and retention factors at the ingredient level [41]; the importance of selecting specific nutrient retention factors is also emphasized. In this report [41] was suggested and then marked by Westenbrink et al. [49] also the use of common retention factors for vitamins and minerals according to the EuroFIR food classification and the cooking methods available in the LanguaL^TM^ Thesaurus (http://www.langual.org).

A Guideline for calculating nutrient content based on the recommendations of EuroFIR AISBL [24] has recently been formulated. This document provides information for different users, especially, to food commercial operators, by including also several components specified for processed foods: list of ingredients; weight of input ingredients; total raw weight of input ingredients; weight of cooked food; food composition data of input ingredients; calculation—content of nutrients in cooked food without using retention factors; retention factors; calculation—content of nutrients in cooked food with retention factors; calculation of energy value and as additional step calculation of water. However, it is recommended to check outputs of a recipe calculation by chemical analysis of a product [24,41].

The most correct method is still under discussion (EuroFIR Food Forum 2017; http://www.eurofir.org/foodforum/). Some researchers from the EuroFIR AISBL association, within the FoodCASE project, are developing a computerized method that takes into account all the procedures discussed to calculate nutrient content of processed foods [50,51].

## 4. Conclusions

This work wants to give an updated overview of the Food Composition Databases, as essential tools in nutrition research for a wide spectrum of applications; currently the main issues to be addressed are the following:

Chemical analysis of foods, recipes, manufactured foods etc. by producing new experimental values with other national collection of composition data, also for traditional and certified foods, must be carried out to increase also the total quality of Food Composition Databases.

Enhance and update regularly the Food Composition Databases by reducing the missing data, to improve also the results of the calculation methods.

Provide and assess specific correction factors, considering the times and appropriate ways of cooking, to ensure a reliable calculation of the composition of complex dishes.

Conduct further studies on specific retention factors to compensate the intrinsic variability of micronutrients and make their calculated values more reliable.

Recipe calculations must be carried out by persons with basic knowledge of food chemistry and appropriate skills for considering the complex changes occurring during processing of foods.

For nutrition labeling, which follows precise procedures but also permits greater tolerance of the nutritional declared values, the calculated data should be considered reasonable and of great utility to the food business operators (Regulation (EU) No. 1169/2011).
